# Context- and Subgroup-Specific Language Changes in Individuals Who Develop PTSD After Trauma

**DOI:** 10.3389/fpsyg.2020.00989

**Published:** 2020-05-15

**Authors:** German Todorov, Karthikeyan Mayilvahanan, Christopher Cain, Catarina Cunha

**Affiliations:** ^1^Emotional Brain Institute, The Nathan Kline Institute for Psychiatric Research, Orangeburg, NY, United States; ^2^Department of Neurobiology and Behavior, School of Medicine, Stony Brook University, Stony Brook, NY, United States; ^3^NYU Langone Health, Department of Child & Adolescent Psychiatry, New York, NY, United States; ^4^The Nathan Kline Institute for Psychiatric Research, Orangeburg, NY, United States

**Keywords:** PTSD, trauma, natural language use analysis, diagnostic tool, screening

## Abstract

Post-traumatic stress disorder (PTSD) is a very common condition with more than 3 million new cases per year in the US alone. The right diagnosis in a timely manner is key to ensuring a prompt treatment that could lead to a full recovery. Unfortunately, avoidance of trauma reminders, social stigma, self-presentation, and self-assessment biases often prevent individuals from seeking timely evaluation, leading to delays in treatment and suboptimal outcomes. Previous studies show that various mental health conditions are associated with distinct patterns of language use. Analyzing language use may also help to avoid response bias in self-reports. In this study, we analyze text data from online forum users, showing that language use differences between PTSD sufferers and controls. In all groups of PTSD sufferers, the usage of singular first-person pronouns was higher and that of plural first-person pronouns was lower than in control groups. However, the analysis of other word categories suggests that subgroups of people with the same mental health disorder (here PTSD) may have salient differences in their language use, particularly in word usage frequencies. Additionally, we show that word usage patterns may vary depending on the type of the text analyzed. Nevertheless, more studies will be needed to increase precision by further examine a variety of text types and different comorbidities. If properly developed, such tools may facilitate earlier PTSD diagnosis, leading to timely support and treatment, which are associated with better outcomes.

## Introduction

Currently, in the US alone, nearly 6 million people are disabled by mental illness. This number rises each day by more than 400. This is not just a health crisis but also an economic one: direct and indirect global economic costs of mental illness were estimated at US$2.5 trillion as of 2010. As reported by the National Center for post-traumatic stress disorder (PTSD), 7–8% of the US population will experience PTSD at some point in their lifetime. To maintain economic and social stability, mental illness must be prevented and treated. In the past few decades, considerable progress has been made in understanding mental illness (Norquist and Hyman, 1999), leading to the development of more effective treatment options. Nonetheless a large portion of this population remains untreated. Nationwide epidemiologic studies show that over half of the people with mental health disorders who would benefit from treatment do not receive it (Regier et al., 1993; Kessler et al., 2001).

Numerous studies have tried to explain why some people with mental illness seek treatment while others do not. In both military and civilian populations, common factors include fear of stigmatization, as well as self-presentation and self-assessment biases (Olfson et al., 1998; Perlick et al., 2001; Link et al., 2008; Ahmedani, 2011; Bonfils et al., 2018).

Another important factor is the inability of the affected individuals to recognize the symptoms (Ahmedani, 2011), which prevents help-seeking and at best delays diagnosis and treatment. Most mental health disorders are much easier to control and reverse if they are diagnosed and treated early. Currently used mental health evaluations, such as the those based on DSM (Diagnostic and Statistical Manual of Mental Disorders), require specialized help that is not always promptly available. However, early and accurate diagnosis is key to facilitating timely and effective treatment that could lead to a full recovery (Acosta et al., 2013; Burnam et al., 2014; Brahm et al., 2016; Jones et al., 2017; Horn and Feder, 2018). In that context, individuals with mental health concerns could benefit from an anonymous and easily accessible self-testing tool that analyzes language usage patterns and correlates such patterns to mental health conditions or risk thereof. While unsuitable for any definitive diagnosis, such a tool may prompt at-risk individuals to seek timely mental health evaluation and care.

Adding to the problem, the evidence from the broadly studied “Halo effect” (definition: the extrapolation of a general impression on evaluations of individual attributes of a person) (Sappenfield, 1971; Cockayne and Samuelson, 1978; Smith, 1988) and Dr. Daniel Kahneman’s and Dr. Amos Tversky’s work show a general assessment bias in the observer, even in highly trained humans (Kahneman, 2003). Already in the 1960s Dr. Hoffman’s and colleagues’ work which was summarized by Dr. Lewis Goldberg, showed that in some situations simple algorithms could outperform clinical specialists in diagnosing diseases (Goldberg, 1968).

To address these issues, researchers in mental health related fields started using implicit or indirect evaluation methods (Pressman and Cohen, 2007; Thoma et al., 2013). In particular, language analysis may help to bypass self-presentation and self-assessment biases common in self reports (Mehl and Pennebaker, 2003; Pennebaker et al., 2003; Pennebaker, 2011). While a wide variety of semantic, syntactic and other language usage patterns may be associated with the state of a person’s mental health, word usage frequencies appear to be the least affected by incidental variations, such as education level. Some word categories appear to be especially significant for assessing an individual’s mental state via language analysis. For instance, function words, such as pronouns, account for only about 1% of all vocabulary words, but represent approximately 15% of the total words used, and appear to be strongly linked to underlying mental processes (Miller, 1995; Tausczik and Pennebaker, 2010). Language usage, including word category frequencies, is affected by a variety of factors, including age (Pennebaker and Stone, 2003), gender (Mehl and Pennebaker, 2003), and personality (Hirsh et al., 2009). However, for certain word categories, the state of an individual’s mental health may have the greatest impact on word usage frequency (Pennebaker, 1993). In fact, the approach to access mental states by analyzing patterns of language usage is supported by a growing body of research in the fields of psychology and neuroscience (Pennebaker et al., 2003). Previous studies using Linguistic Inquiry and Word Count (LIWC) have found that it is possible to characterize depression and other mental states through analyzing natural language use (Tausczik and Pennebaker, 2010; Booker et al., 2018; Kaplow et al., 2018; Tackman et al., 2018). For example, Stirman and Pennebaker showed that suicidal poets displayed a higher usage of first person pronouns in their texts and less first-plural pronouns than non-suicidal poets (Stirman and Pennebaker, 2001). Rude, Gortner, and Pennebaker demonstrated that the language of depressed students has a higher frequency of first person singular pronouns and negative emotion words, and a lower frequency of positive emotion words, compared to students that never experienced depression (Rude et al., 2004). Together, these results support the social engagement/disengagement model of depression (Fisher and Chon, 1989), and Pyszcynski and Greenberg’s self-awareness theory of depression (Pyszczynski and Greenberg, 1987). Furthermore, clinicians have observed higher use of first singular pronouns in depressed patients (Bucci and Freedman, 1981; Weintraub et al., 1981), and lower second-person pronoun usage, which may reflect a decreased sense of community (Simmons et al., 2005).

People with PTSD were shown to use significantly fewer second-person pronouns (Coppersmith et al., 2014b). Additionally, studies have shown differences in the use of causal and cognitive words during recalls of negative events (Boals and Klein, 2005), which may be an indicator of the attempt to rationalize and resolve traumatic experiences (Kross and Ayduk, 2008). The analysis of additional linguistic markers, such as the use of negative emotion words, cognition words, and insight words predicted the future mental health of college students who wrote about traumatic events (Pennebaker, 2001). Furthermore, the presence of words relating to death and dying was an indicator of treatment-resistant PTSD (Alvarez-Conrad et al., 2001). Consequently, the analysis of linguistic elements in different text types could be crucial for understanding cognitive mechanisms associated with trauma and may hold a valuable potential to diagnose and predict PTSD symptoms and subtypes. If properly developed, such technology could help individuals to self-test and public health organizations to screen for possible mental health conditions and prompt further evaluation when warranted, potentially preventing disorders from becoming chronic, debilitating, and difficult to treat.

While language analysis programs could be an effective means for tentative self-diagnosis and mental health screening, the development of such programs is impeded by the gaps and inconsistencies in our current understanding of the links between language usage and mental illness. The few existing language analysis programs are based on data from generalized mentally ill populations that commingle otherwise fairly distinct subgroups. This often results in disregarding salient individual differences and may lead to misdiagnosis.

A major hurdle for developing more specific and reliable language based diagnostic tools has been the lack of variety in the available language data. With the growth of social media, internet forums are shown to present a useful source of naturalistic writing from people that use these forums as an anonymous and inexpensive self-help tool, especially for stigmatizing mental health illnesses such as PTSD (Baker et al., 2003; Lampe et al., 2003; Berger et al., 2005). Also, a practically useful and versatile pre-diagnostic and screening language analysis tool should produce salient results even from relatively short text samples common in social media and modern communications. Recent studies show that using an automated word counting approach is an efficient way to characterize the language of online groups (Lyons et al., 2006). Patterns of emotional and cognitive expression in internet support groups were used to research depression and other affective disorders (Houston et al., 2002; Griffiths et al., 2009), and as a predictor of future mental health in cancer patients and recovering anorexics (Lieberman and Goldstein, 2006; Lyons et al., 2006).

We hypothesize that there are salient language use characteristics throughout different text samples from people affected by PTSD. The goal of this project is to identify significant indicators of the common PTSD-related pathologies, as it could help to develop novel diagnostic tools for broad screening of the general population.

## Materials and Methods

### Data Collection and Categorization

In this study, we analyze text data from online forum users and differentiate between people that have PTSD from those that do not. Data for this study are composed of text samples from public forums collected and screened according to previously described procedures (Coppersmith et al., 2014a). Forums users often discuss their health for various reasons, such as to seek support or advice. More specifically to mental health, users may choose an anonymous forum due to the social stigma associated with mental illness. Many forum users describe their diagnosis in a large variety of mental health conditions (Coppersmith et al., 2015). In this study we focused on PTSD. A human editor assessed each description of diagnosis and removed quotes or other disingenuous text sections (Example of disingenuous statements of diagnosis: “Omg I just messed up my makeup! I’m literally crying I have PTSD” – anonymous).

To ensure that each included forum user has a sufficient amount of data, we ensured that each user had at least 100 words, however over 80% had between 200 and 500 words.

Ideally, age and gender should be controlled when performing mental health research. A small amount of studies have taken matched samples into consideration for example, by examining a specific subgroup of population such, as college students (Rude et al., 2004). In order to have age- and gender-matched groups, we analyzed each text sample and its language as described in previously published studies (Sap et al., 2014). To obtain our final data set for each PTSD forum user, we determined or estimated the gender and age using the user profile self-description and by analyzing the user’s other posts. When compiling control groups, we selected the text samples from the users with matching estimated gender and the closest estimated age.

### Data

Text samples for the PTSD group were collected from various internet forums dedicated to PTSD^1^ (Table 1). A large number of potentially suitable text samples were manually screened and either discarded or sorted into the groups of interest-based on the content of the excerpt itself, forum thread context, and other available anonymous information. Previous studies often compare data from people affected by PTSD vs. healthy people/general population data. In our study, we also used general population data as a control group. However, it is also important to compare PTSD data to text samples from traumatized individuals that didn’t develop PTSD to control for trauma-related word patterns unrelated to PTSD. This also allows for the possible detection of resilience-related word patterns. Therefore, our first control group consists of data from firefighter forums, where users discuss work, daily life and extreme/traumatic situations, yet, are not suffering from PTSD (Table 1). Our second control group consists of general population data (Table 1).

**TABLE 1:** Description of PTSD subgroups and control groups for language use analysis.

Subgroup	Number of text samples (excerpts)
Individuals with PTSD sharing daily events	21
Individuals who suffered trauma within last 12 month and are at risk for PTSD	16
Individuals who suffered trauma years ago and developed PTSD	21
Military veterans, police officers, firefighters with PTSD	19
Firefighters without PTSD (Control 1)	19
General population (Control 2)	26

*Datasets used for study (total *n* = 122).*

We also distinguished between narrative related to the trauma and daily life event narratives (Table 1). Forums have various discussion group categorizations. For *“trauma narratives”* we choose text that discussed the trauma event explicitly. We collected text samples for *“daily life narratives”* from journal entry discussion groups where forum users shared their daily lives with other users but made no explicit mention of trauma events (Table 1).

### Analysis

We used word usage frequency analysis conceptually similar to that in LIWC methodology and program (Pennebaker et al., 2003). LIWC was shown to be effective in detecting a number of psychologically salient language usage patterns. We developed a custom software program that extended LIWC approach by combining it with character language models (CLMs) for additional word matching features, as well as additional and/or modified word categories tailored to PTSD. This provided a score even for very short texts (McNamee, 2004). Word matching included pattern matching whole words, roots, salient word parts, simple stemming, split verb/expression stemming, and others.

Based on literature (Pennebaker, 1993, 2001; Stirman and Pennebaker, 2001; Pennebaker and Stone, 2003; Kenardy et al., 2007; Tausczik and Pennebaker, 2010; Badger et al., 2011; Jaeger et al., 2014; Mott et al., 2015; Knutsen and Jensen, 2017; Westerman et al., 2017; Rometsch-Ogioun El Sount et al., 2018) and text screening, the following word categories were determined to be potentially salient for PTSD and/or depression and were used in this study:

• Singular first-person pronouns (related to self only);
• Plural first-person pronouns (related to group including self);
• Words positively correlated with depression (Stirman and Pennebaker, 2001; Pennebaker et al., 2003; Rude et al., 2004; Tausczik and Pennebaker, 2010; Baddeley et al., 2011; Mowery et al., 2017);
• Negative emotions;
• Mortality, death, and dying;
• Indicators of cognitive complexity;
• Words indicating causative relationships.

For each word category and population group, a standard set of usage frequency statistical data was calculated (using *stats* and *stats-lite* npm software modules), including mean, median, variance, standard deviation, and percentile distribution. Statistical significance of group differences was calculated using *t*-test, ANOVA, and *post hoc* Tukey test. For each user, we scored each text based on the character n-grams in the text with the CLMs for the condition. This method followed previous work on predicting mental health in social media (Coppersmith et al., 2014a).

This study is an analysis of existing, de-identified and publicly available data. No sensitive information was collected, and the study data is completely anonymous. As by regulation of §46.104, if the project does not include any interaction or intervention with human subjects or include any access to identifiable private information, then the project does not require IRB review and is exempt.

## Results

The present study employed a computerized text analysis to examine language usage patterns in people affected by PTSD. We observed differences in linguistic markers in posts of similar word count written by different groups of people affected by PTSD and two control groups.

### Texts From People With PTSD Whose Trauma Occurred Years Ago Differ From Those Written by PTSD Sufferers With Recent Trauma

**FIGURE 1:**
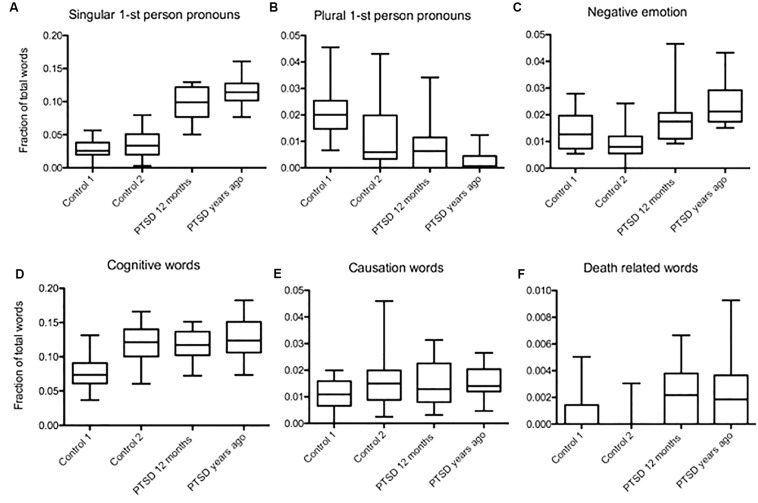
Word usage patterns in texts related to trauma that occurred years ago and recent trauma. **(A)** Higher frequency of singular first-person pronouns in both PTSD groups compared to controls (Mdiff = 0.068, Mdiff = 0.087; Mdiff = 0.061, Mdiff = 0.08, *p* < 0.001). **(B)** Lower frequency in plural first-person pronouns in both PTSD groups compared to control 1, the lowest one found in the group whose trauma occurred years ago (Mdiff = 0.014, Mdiff = 0.019, *p* < 0.001). Only PTSD group whose trauma occurred years ago showed a significant difference in plural first- person pronoun usage compared to control 2 (Mdiff = 0.006; *p* > 0.05; Mdiff = 0.01, *p* < 0.05). **(C)** Usage of negative emotion words was only different from control 1 in the PTSD group where trauma occurred years ago (Mdiff = 0.0097, *p* < 0.001). Compared to control 2, both PTSD groups showed a significantly higher usage of negative emotion words (Mdiff = 0.0096, *p* < 0.001; Mdiff = 0.0149, *p* < 0.001). **(D)** Cognitive words were more frequent in both PTSD groups compared to control 1, but not control 2 (Mdiff = 0.038, Mdiff = 0.049, *p* < 0.001; Mdiff = 0.003, Mdiff = 0.007, *p* > 0.05). **(E)** There was no significant difference in causation word usage between all three PTSD groups and/or controls (*p* > 0.05). **(F)** Death related word usage was higher in both PTSD groups compared to control 2 (Mdiff = 0.0019, *p* < 0.05; Mdiff = 0.0023, *p* < 0.001).

We distinguished between people whose trauma occurred recently (within 12 months) and those who experienced trauma years ago, including childhood trauma. In both PTSD groups singular first-person pronoun usage was higher than in the two control groups (Mdiff = 0.068, Mdiff = 0.087; Mdiff = 0.061, Mdiff = 0.08, *p* < 0.001, Figure 1A). First person plural pronouns were lower in frequency in both PTSD groups compared to control 1, although in the group where trauma occurred years ago the usage was the lowest (Mdiff = 0.014, Mdiff = 0.019, *p* < 0.001, Figure 1B). Compared to control 2, only text from the PTSD years ago group showed a significant difference in plural first-person pronoun usage (Mdiff = 0.006; *p* > 0.05; Mdiff = 0.01, *p* < 0.05, Figure 1B). Compared to control 1, the usage of negative emotion words was only different in the PTSD group where trauma occurred years ago (Mdiff = 0.0097, *p* < 0.001, Figure 1C). However, compared to control 2, both PTSD groups showed a significantly higher usage of negative emotion words (Mdiff = 0.0096, *p* < 0.001; Mdiff = 0.0149, *p* < 0.001, Figure 1C). The usage of cognitive words was higher in both PTSD groups compared to control 1, but not control 2 (Mdiff = 0.038, Mdiff = 0.049, *p* < 0.001; Mdiff = 0.003, Mdiff = 0.007, *p* > 0.05, Figure 1D). In contrast to the previously published data (Jaeger et al., 2014), we could not detect a significant difference in causation words (*p* > 0.05, Figure 1E). Death-related word usage was higher in both PTSD groups compared to control 2 (Mdiff = 0.0019, *p* < 0.05; Mdiff = 0.0023, *p* < 0.001, Figure 1F).

### Language Usage Differences Detected Comparing People With PTSD Whose Trauma Occurred in a Work Setting to PTSD Sufferers With Personal Life Related Trauma

**FIGURE 2:**
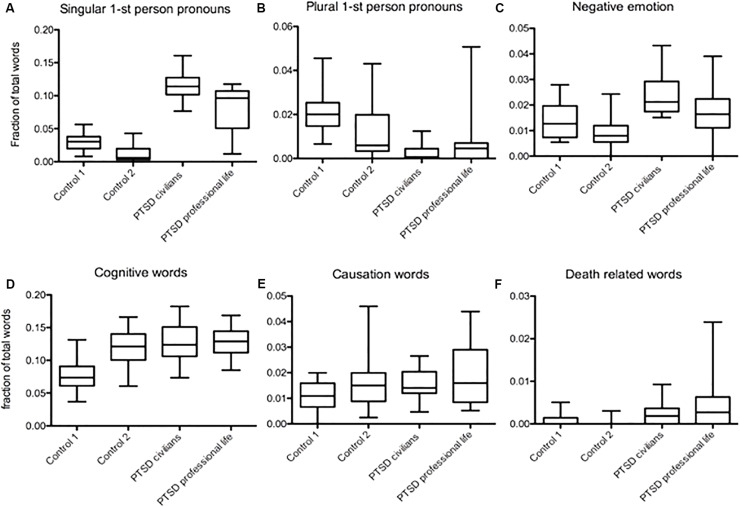
Word patterns in texts from people PTSD sufferers whose trauma occurred in a work setting compared to personal life related trauma. **(A)** Higher frequency of singular first-person pronouns in both PTSD groups compared to both controls, the highest found in civilians with PTSD (Mdiff = 0.086, Mdiff = 0.052, *p* < 0.001; Mdiff = 0.102, Mdiff = 0.0683, *p* < 0.001). **(B)** Lower frequency in plural first-person pronouns in both PTSD groups compared to control 1, but compared to control 2 only PTSD in civilians show a significant difference (Mdiff = 0.019, Mdiff = 0.015, *p* < 0.001; Mdiff = 0.0103, *p* < 0.01, Mdiff = 0.0068, *p* > 0.05). **(C)** Both PTSD groups had a higher frequency of negative emotion words but only compared to control 2 (Mdiff = 0.0149, *p* < 0.001; Mdiff = 0.0086, *p* < 0.05). Compared to control 1 only civilians showed a difference in negative emotion word usage (Mdiff = 0.0097, *p* < 0.05). **(D)** Higher usage frequency of cognitive words in both PTSD groups compared to control 1 (Mdiff = 0.049, *p* < 0.001; Mdiff = 0.0503, *p* < 0.001) but not to control 2 (*p* > 0.05). **(E)** There was no significant difference in causation word usage between the two PTSD groups and/or controls (*p* > 0.05). **(F)** Death related word occurrence was higher in the professional life related trauma group than in both controls, the difference being greater comparing to control 2 (Mdiff = 0.0033, *p* < 0.05; Mdiff = 0.004, *p* < 0.001).

We identified a set of variables in language usage in two different groups of PTSD, (1) people who went through trauma at work, including veterans, police officers, and firefighters (professional life), and (2) people that experienced trauma in their personal lives (civilians). The usage of singular first-person pronouns was higher in civilians, but also high in the professional group compared to both controls (Mdiff = 0.086, Mdiff = 0.052, *p* < 0.001; Mdiff = 0.102, Mdiff = 0.0683, *p* < 0.001, Figure 2A). In both groups, plural first-person pronouns occurred significantly less frequently than in the control 1. However, compared to control 2, only civilians affected by PTSD had a lower occurrence of plural first-person pronouns (Mdiff = 0.019, Mdiff = 0.015, *p* < 0.001; Mdiff = 0.0103, *p* < 0.01, Mdiff = 0.0068, *p* > 0.05, Figure 2B). Negative emotion words were used more frequently in civilians compared to control 1 (Mdiff = 0.0097, *p* < 0.05, Figure 2C). Compared to control 2, both PTSD groups had a higher frequency of negative emotion (Mdiff = 0.0149, *p* < 0.001; Mdiff = 0.0086, *p* < 0.05, Figure 2C). Analysis of cognitive words showed a higher usage frequency in both PTSD groups than in control 1 (Mdiff = 0.049, *p* < 0.001; Mdiff = 0.0503, *p* < 0.001, Figure 2D), but no difference compared to control 2 (*p* > 0.05, Figure 2D). There was no significant difference in causation word usage between the two PTSD groups and controls (*p* > 0.05, Figure 2E). Death-related word occurrence was higher in the professional life-related trauma group than in both controls with the difference being greater when compared to control 2 (Mdiff = 0.0033, *p* < 0.05; Mdiff = 0.004, *p* < 0.001, Figure 2F).

### Word Patterns Vary in Different Text Types From People With PTSD: Comparing Daily Life Narratives to Trauma Narratives

**FIGURE 3:**
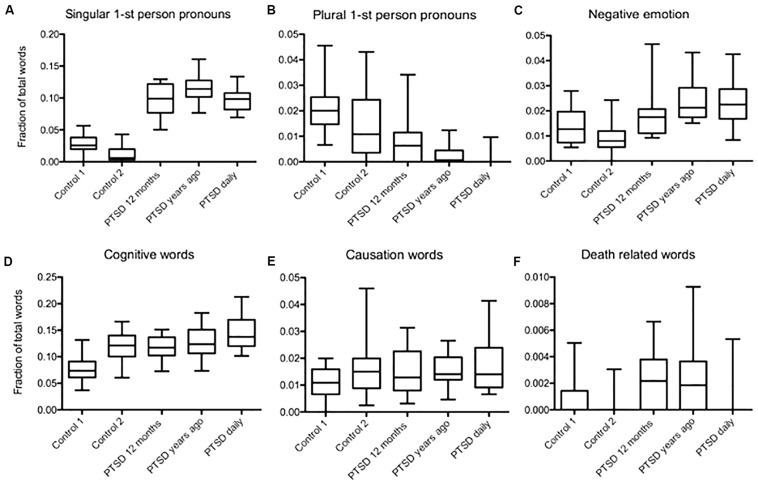
Word usage patterns in daily narratives and trauma related narratives of PTDS sufferers. **(A)** Higher frequency of singular first-person pronouns in both PTSD related text types compared to controls, the highest one found in the trauma narratives by individuals whose trauma occurred years ago (Mdiff = 0.067, Mdiff = 0.087, Mdiff = 0.069, *p* < 0.001; Mdiff = 0.0827, Mdiff = 0.102, Mdiff = 0.0844, *p* < 0.001). **(B)** Lower usage of plural first-person pronouns in daily narratives compared to recent trauma related text occurrence (Mdiff = 0.0063, *p* < 0.05). However, all three PTSD groups used fewer plural first-person pronouns than control 1, but compared to control 2 only the recent trauma group and daily narratives showed a significant difference (Mdiff = 0.019, Mdiff = 0.014, Mdiff = 0.02, *p* < 0.001; Mdiff = 0.0069, Mdiff = 0.0133, *p* < 0.001, Mdiff = 0.012, *p* > 0.05). **(C)** Negative emotion word frequency was highest in daily narratives, but it was also high compared to controls in the reports of trauma that occurred years ago (Mdiff = 0.008, *p* < 0.05; Mdiff = 0.0135, *p* < 0.05; Mdiff = 0.009, *p* < 0.05, Mdiff = 0.015, *p* > 0.05). **(D)** Cognitive word frequency was the highest in daily narratives of PTSD sufferers. In all three PTSD groups, it was higher than in control 1, but not significantly higher than in control 2 (Mdiff = 0.039, Mdiff = 0.049, Mdiff = 0.066, *p* < 0.001; *p* > 0.05). **(E)** There was no significant difference in causation word usage between PTSD groups and controls (*p* > 0.05). **(F)** Death related word type usage was increased in both trauma text types compared to only control 2 but not compared to control 1, daily narratives didn’t differ significantly compared to both controls (Mdiff = 0.0019, Mdiff = 0.0023, *p* < 0.05; *p* > 0.05; *p* > 0.05).

We compared trauma narratives and daily life narratives from people with PTSD. In both PTSD related text types, singular first-person pronouns occurred more often than in controls. We found the highest frequency in the trauma narratives written by individuals whose trauma occurred years ago (Mdiff = 0.067, Mdiff = 0.087, Mdiff = 0.069, *p* < 0.001; Mdiff = 0.0827, Mdiff = 0.102, Mdiff = 0.0844, *p* < 0.001, Figure 3A). We detected a significantly lower usage of plural first-person pronouns in daily narratives compared to recent trauma narratives (Mdiff = 0.0063, *p* < 0.05, Figure 3B). Nevertheless, all three PTSD groups used fewer plural first-person pronouns than control 1, but compared to control 2 only the recent trauma group and daily narratives showed a significant difference (Mdiff = 0.019, Mdiff = 0.014, Mdiff = 0.02, *p* < 0.001; Mdiff = 0.0069, Mdiff = 0.0133, *p* < 0.001, Mdiff = 0.012, *p* > 0.05, Figure 3B). Negative emotion word usage was the highest in the daily narratives, but it was also higher than the controls in the narratives where trauma had occurred years ago. There was no significant difference in negative emotion word usage between recent trauma related texts and controls (Mdiff = 0.008, *p* < 0.05; Mdiff = 0.0135, *p* < 0.05; Mdiff = 0.009, *p* < 0.05, Mdiff = 0.015, *p* > 0.05, Figure 3C). Cognitive word frequency was the highest in daily narratives of PTSD sufferers. In all three PTSD groups, it was higher than in control 1, but not significantly higher than in control 2 (Mdiff = 0.039, Mdiff = 0.049, Mdiff = 0.066, *p* < 0.001; *p* > 0.05, Figure 3D). There was no significant difference in causation word usage between PTSD groups and controls (*p* > 0.05, Figure 3E). Death-related word usage was increased in both trauma text types compared to control 2, but not compared to control 1 (Mdiff = 0.0019, Mdiff = 0.0023, *p* < 0.05; *p* > 0.05; *p* > 0.05, Figure 3F).

### Language Analysis Suggests Positive but Variable Correlation Between Depression and PTSD in Different Groups of Affected People

In this section of our study, we used a recently developed procedure for analyzing depression through language usage, which is based on previous studies (Stirman and Pennebaker, 2001).

**FIGURE 4:**
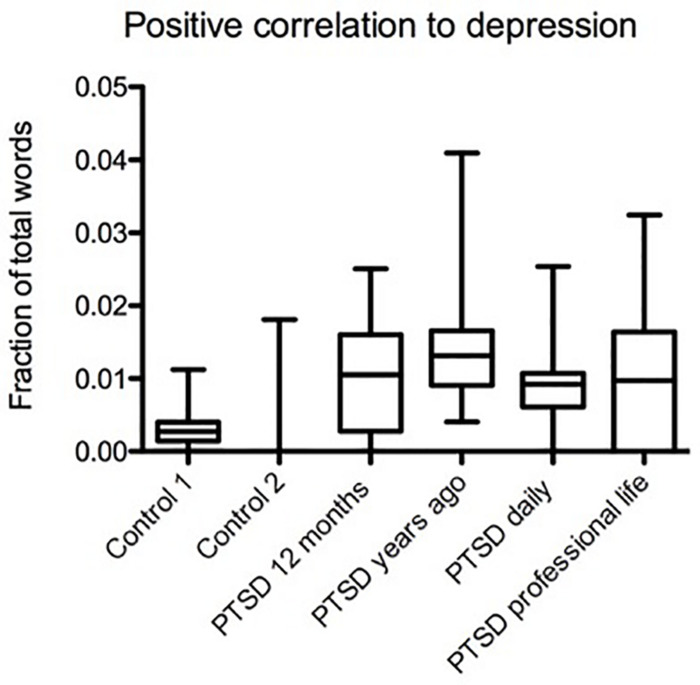
Variable language-based positive correlation between depression and PTSD in different groups. The highest usage frequency of depression-correlated words was in PTSD sufferers who experienced trauma years ago, the difference being statistically significant compared to control 1, but not control 2 (Mdiff = 0.011, Mdiff = 0.007, *p* < 0.001, *p* > 0.05). The second highest usage frequency of depression-correlated words was in the PTSD group with professional life related trauma (Mdiff = 0.007, Mdiff = 0.008, *p* > 0.05, *p* < 0.05). Daily narratives of PTSD sufferers had more depression-correlated words than daily narratives of control 2, a general population sample (Mdiff = 0.0077, *p* > 0.05).

We found the highest usage frequency of words positively correlated with depression in PTSD sufferers who experienced trauma years ago. However, the difference was only statistically significant compared to control 1, not to control 2 (Mdiff = 0.011, *p* < 0.001; *p* > 0.05, Figure 4). The second highest usage frequency of depression-correlated words was found in the PTSD group with professional life-related trauma (Mdiff = 0.007, *p* < 0.05; Mdiff = 0.009, *p* < 0.05, Figure 4). However, depression-correlated word usage was somewhat elevated in all PTSD groups we analyzed. Comparison of daily narratives from PTSD sufferers and daily narratives from the general population (control 2) points to an overall comorbidity of PTSD and depression as indicated by word usage (Mdiff = 0.0077, *p* < 0.01, Figure 4).

## Discussion

Our analysis, based on text samples from PTSD online forums, shows that the language use in PTSD appears to vary from that of controls in a number of word categories, including first and third person pronouns, negative emotion words, death- and dying-related words, as well as words indicating cognitive complexity (Table 2). Furthermore, our data shows word usage differences between distinct groups of PTSD sufferers. We also found that word usage patterns are affected by the type of text. Overall, our results demonstrate the importance of taking into account additional factors, such as population subtypes and text types when analyzing language usage as a reflection of mental states and their pathologies. Considering the results of a study examining predictors of PTSD after burn injuries, the lack of psychological support seems to be a stronger predictor of PTSD than the severity or nature of the trauma (Perry et al., 1992), the development of feasibly accurate and accessible analytical tools facilitating early diagnosis appears to have value for both prevention and effective treatment of PTSD. Nonetheless, our research is limited by using online forum data set types, and more studies will be needed to increase precision by further examination of variable data resources, and a number of important or conflicting variables, such as specific patient subgroups, situational background, and co-morbidities.

**TABLE 2:** Summary of data analysis of language use in PTSD compared to controls.

Group	Singular 1-st person pronouns	Plural 1-st person pronouns	Negative emotion	Cognitive words	Causation words	Death related words	Depression correlation
PTSD 12 months	Increased	Low	Regular	Regular	Regular	Increased	Increased
PTSD years ago	Increased	Lower	Increased	Regular	Regular	Increased	Increased
PTSD daily	Increased	Lowest	Increased	Slight increase	Regular	Regular	Increased
PTSD professional	Increased	Lower	Increased	Regular	Regular	Increased	Less increased

We found the greatest differences in the usage of first-person pronouns. All groups of PTSD sufferers, regardless of text type, had a markedly higher usage of singular first-person pronouns than the controls. This suggests an increased focus on oneself (Campbell and Pennebaker, 2003; Pennebaker et al., 2003), possibly at the expense of the focus on, and collaboration with, others. In fact, we found markedly lower usage of the plural first-person pronouns in text types of daily narratives and in civilians with PTSD whose trauma occurred years ago. On the other hand, individuals with recent trauma had an only mild-to-moderate decrease in plural first-person pronoun usage, which may reflect the dynamics of early stage PTSD development. Low occurrence of plural first-person pronouns in PTSD texts, particularly in long-standing PTSD, may also be an indicator of comorbidity with depression and a predictor of suicidal tendencies, which is consistent with the social integration model of suicide (Stirman and Pennebaker, 2001).

The observed pattern of markedly higher singular first-person pronoun usage, especially when combined with lower frequency of the plural pronouns, may be salient, not only for detecting existing PTSD but also, and perhaps more importantly, for assessing the risk of developing PTSD in individuals with recent trauma, i.e., during the time window when intervention and treatment tend to be the most effective. Studies show that the type of trauma is not, by itself, a reliable predictor of PTSD, whereas certain characteristic signs and symptoms, such as persistent avoidance, are indicative (Dancu et al., 1996; Panasetis and Bryant, 2003; Briere et al., 2005). In that context, we should note the earlier findings that higher usage of singular first-person pronouns is associated with greater culpability and shame, which are predictive of developing PTSD during the first year post-trauma (Kubany et al., 2003; Negrao et al., 2005). Also, high singular first-person pronoun use in PTSD groups may be associated with trauma-related persistent dissociation, which is a strong predictor of PTSD (Briere et al., 2005). Overall, the observed pronoun usage patterns appear to have value for detecting both PTSD risk and onset.

We also found a moderately higher use of negative emotion words in all PTSD groups relative to controls. This word category is known to strongly correlate with the symptoms of depression. Notably, among PTSD groups, negative emotion words were higher in the groups with long-standing PTSD and lower (but still elevated relative to control) in the recent trauma group. We also analyzed the usage of depression-correlated words, a composite category comprising a representative sample of words commonly correlating with depression. We found that the usage of depression-correlated words was increased in all PTSD groups, but more dramatically in the group with long-standing PTSD. Our findings are consistent with prior research indicating that PTSD comorbidity with depression is common (ca. 50%) and tends to occur in people with more severe and persistent forms of PTSD (Flory and Yehuda, 2015). This hypothesis appears to be further supported by our data showing higher death-related word usage in the PTSD groups with either a very recent trauma or a trauma that occurred years ago. However, while the usage frequencies of death-related words in these groups are elevated to similar levels, the reasons may be different. In the recent trauma group, the increase in death-related word usage may reflect an acute reaction and processing of the recent trauma, especially if such trauma involved witnessing fatalities. In the group who experienced trauma far in the past, it may reflect a higher prevalence of severe, chronic depression that is linked to a higher risk of suicide (Stirman and Pennebaker, 2001; Indu et al., 2017; Ophuis et al., 2018; Dong et al., 2019). Proper interpretation of such group-related distinctions may improve the accuracy of pre-diagnostic evaluation and assist in guiding therapeutic intervention. Furthermore, it would be critical to recognize the subgroup of PTSD sufferers showing comorbidity with depression, because they generally have a higher level of neurocognitive distress (Blanchard et al., 1998; Nijdam et al., 2013; Flory and Yehuda, 2015) and are at a greater risk of suicide than people with PTSD alone (Campbell et al., 2007; Ramsawh et al., 2014). It would be also crucial to determine if current treatment options are effective in people showing comorbidity of PTSD with depression.

There are a number of different comorbidities that we didn’t analyze in this study; for example, substance use disorders, and other anxiety disorders. However, this overlap leads in many cases to diagnostic confusion and especially to the underdiagnosis of PTSD when trauma histories were not collected (Brady et al., 2000). It will be necessary to further research these various comorbidities in future studies. In our cognitive mechanism word analysis, we distinguished between causation and cognitive words. There were no significant differences in causation word frequencies between PTSD groups and controls. Cognitive word usage was increased in all PTSD groups relative to control 1 but was similar to control 2. This difference may be the most meaningful for the professional-life related PTSD group for which control 1 (firefighters without PTSD) is a better match. Cognitive word usage was somewhat higher in the daily narratives of PTSD sufferers than in trauma narratives, which is in line with previous research (Rubin, 2011).

It is essential for both research and clinical practice to be able to accurately assess psychological well-being in order to identify individuals who are at risk or have developed PTSD after trauma. Within that population, it is also critically important to detect cases at higher risk for suicide in view of a known relationship between traumatic experiences and suicidal behaviors (Knox, 2008).

In a future study, it would be interesting to conduct the analysis of the data sets from the perspective of sexual battery/abuse-related trauma, especially for the individuals who suffered trauma years ago and developed PTSD. However, we excluded this perspective from the present study because we wanted to establish salient differences across broader populations and different trauma backgrounds in order to develop an easy-to-use PTSD pre-diagnostic tool that did not require too much information from the traumatized person, since such requirement could hinder engagement and timely diagnosis.

Explicit screening methods are often deficient due to biases in self-assessment, self-presentation, and self-reporting, as well as avoidance due to lack of anonymity. Implicit screening methods, such as language analysis, have the capability to increase accuracy and reliability used either alone or in conjunction with explicit screening. Furthermore, this type of screening can provide anonymous self-prediagnosis without the risk of social stigma, thus reducing the obstacles to seeking immediate care. Facilitating prompt care could decrease PTSD rates and/or severity in people who have suffered a recent trauma. Further studies should help improve the accuracy and practical utility of language usage analysis for PTSD and other conditions. Such studies may involve further examining a variety of salient or confounding factors, such as different patient subgroups, situational context, co-morbid conditions, and variable dataset resources. Analyzing language use patterns other than word frequencies, such as syntactic and semantic structures, may yield additional insights. Overall, as a supplementary tool, language analysis has an advantage of being economical and time-efficient and also provides a convenient option for anonymous self-prediagnosis and screening.

## Data Availability Statement

The data that support the findings of this study are available from the corresponding author (GT), upon reasonable request.

## Ethics Statement

This study is an analysis of existing, de-identified, and publicly available data. No sensitive information was collected, and the study data is completely anonymous. As by regulation of §46.104, if the project does not include any interaction or intervention with human subjects or include any access to identifiable private information, then the project does not require IRB review and is exempt.

## Author Contributions

CCu and GT conceived of the presented idea. GT developed the theory and performed the computations. CCu and CCa verified the analytical methods. KM assisted GT. All authors discussed the results and contributed to the final manuscript.

## Conflict of Interest

The authors declare that the research was conducted in the absence of any commercial or financial relationships that could be construed as a potential conflict of interest.

## Footnotes

1 https://www.myptsd.com/forums/
